# Dysregulated nicotinamide adenine dinucleotide metabolome in patients hospitalized with COVID‐19

**DOI:** 10.1111/acel.14326

**Published:** 2024-10-01

**Authors:** Rodrigo J. Valderrábano, Benjamin Wipper, Karol Mateusz Pencina, Marie Migaud, Yili Valentine Shang, Nancy K. Latham, Monty Montano, James M. Cunningham, Lauren Wilson, Liming Peng, Yusnie Memish‐Beleva, Avantika Bhargava, Pamela M. Swain, Phoebe Lehman, Siva Lavu, David J. Livingston, Shalender Bhasin

**Affiliations:** ^1^ Research Program in Men's Health: Aging and Metabolism, Boston Claude D. Pepper Older Americans Independence Center Brigham and Women's Hospital, Harvard Medical School Boston Massachusetts USA; ^2^ Department of Pharmacology, Mitchell Cancer Institute University of South Alabama Mobile Alabama USA; ^3^ Division of Hematology, Brigham and Women's Hospital Harvard Medical School Boston Massachusetts USA; ^4^ Metro International Biotech Worcester Massachusetts USA

**Keywords:** 1‐methylnicotinamide, 2PY, NAD^+^ augmentation in COVID‐19, NAD^+^ metabolites, NAD^+^ turnover, nicotinamide, SARS‐CoV‐2 infection

## Abstract

Nicotinamide adenine dinucleotide (NAD^+^) depletion has been postulated as a contributor to the severity of COVID‐19; however, no study has prospectively characterized NAD^+^ and its metabolites in relation to disease severity in patients with COVID‐19. We measured NAD^+^ and its metabolites in 56 hospitalized patients with COVID‐19 and in two control groups without COVID‐19: (1) 31 age‐ and sex‐matched adults with comorbidities, and (2) 30 adults without comorbidities. Blood NAD^+^ concentrations in COVID‐19 group were only slightly lower than in the control groups (*p* < 0.05); however, plasma 1‐methylnicotinamide concentrations were significantly higher in patients with COVID‐19 (439.7 ng/mL, 95% CI: 234.0, 645.4 ng/mL) than in age‐ and sex‐matched controls (44.5 ng/mL, 95% CI: 15.6, 73.4) and in healthy controls (18.1 ng/mL, 95% CI 15.4, 20.8; *p* < 0.001 for each comparison). Plasma nicotinamide concentrations were also higher in COVID‐19 group and in controls with comorbidities than in healthy control group. Plasma concentrations of 2‐methyl‐2‐pyridone‐5‐carboxamide (2‐PY), but not NAD^+^, were significantly associated with increased risk of death (HR = 3.65; 95% CI 1.09, 12.2; *p* = 0.036) and escalation in level of care (HR = 2.90, 95% CI 1.01, 8.38, *p* = 0.049). RNAseq and RTqPCR analyses of PBMC mRNA found upregulation of multiple genes involved in NAD^+^ synthesis as well as degradation, and dysregulation of NAD^+^‐dependent processes including immune response, DNA repair, metabolism, apoptosis/autophagy, redox reactions, and mitochondrial function. Blood NAD^+^ concentrations are modestly reduced in COVID‐19; however, NAD^+^ turnover is substantially increased with upregulation of genes involved in both NAD^+^ biosynthesis and degradation, supporting the rationale for NAD+ augmentation to attenuate disease severity.

## Graphical abstract

Whole blood NAD^+^ levels were only modestly reduced in patients hospitalized with COVID‐19 but the circulating levels of NAD^+^ metabolites, 1‐methylnicotinamide and nicotinamide were markedly elevated. The expression levels of NAD consuming/degrading enzymes as well as of enzymes involved in NAD synthesis were markedly upregulated consistent with increased NAD^+^ turnover. The circulating levels of NAD^+^ metabolite, 2‐PY were associated with risk of death and transfer to intensive care.

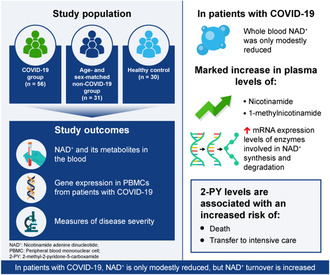

**Abbreviations**
2‐PY: N‐methyl‐2‐pyridone‐5‐carboxamide
4‐PYR: 4‐Pyridone‐3‐Carboxamide Riboside
DEGs: differentially expressed genes
GSEA: gene set enrichment analysis
HPRT: hypoxanthine phosphoribosyl transferase 1
IL‐6: interleukin 6 (IL‐6)
LC‐MS/MS: liquid chromatography tandem mass spectrometry
Me‐NAM: 1‐methylnicotinamide
NAD: nicotinamide adenine dinucleotide
NAM: nicotinamide
PARP: poly (ADP‐ribose) polymerase
PBMC: peripheral blood mononuclear cells

## 1 INTRODUCTION

Coronavirus disease 2019 (COVID‐19) is a potentially life‐threatening illness caused by the severe acquired respiratory syndrome coronavirus 2 (SARS‐CoV‐2) (Bhatraju et al., 2020; Cummings et al., 2020; Wu & McGoogan, 2020). The inhaled SARS‐CoV‐2 binds initially to the epithelial cells in the nasal cavity using ACE2 as the main cell surface receptor, with additional facilitation of cellular entry by TMPRSS2 (Hoffmann et al., 2020; Mason, 2020). The virus propagates and preferentially infects the alveolar type II cells in the lungs that undergo apoptosis thereby releasing viral particles and inciting an inflammatory response, neutrophil and fluid extravasation, increasing vascular permeability, and cell death (Mason, 2020). A majority of people infected with the SARS‐CoV‐2 remain asymptomatic or suffer from only a mild acute respiratory illness; however, a subset of patients infected with the SARS‐CoV‐2 develop a more severe illness that can progress to acute respiratory distress syndrome, multi‐organ failure, and even death (Bhatraju et al., 2020; Cummings et al., 2020; Wu & McGoogan, 2020).

A growing body of data suggests that a heightened inflammatory response and the excessive release of cytokines in patients with COVID‐19 is involved in the development of acute respiratory distress syndrome and multi‐organ failure, and is a major contributor to COVID‐19 mortality (Chen et al., 2020; Mehta et al., 2020; Pedersen & Ho, 2020; Qin et al., 2020). This pathophysiologic cascade, characterized by an aggressive inflammatory response and cytokine release leading to multi‐organ failure, is similar to that observed in severe cases of other respiratory viral diseases, such as SARS, MERS, and influenza (Liu et al., 2016; Ramos & Fernandez‐Sesma, 2015).

NAD^+^ serves as a co‐factor for families of enzymes such as poly (ADP‐ribose) polymerases (PARPs), sirtuins, CD38, BST1, and SARM1 that are involved in innate immune response (Brady et al., 2019; Hogan et al., 2019; Li et al., 2017). NAD^+^ plays an important role in the signaling mechanisms that regulate the NLRP3 inflammasome, NF‐kB signaling, cytokine production, stress granule formation, antiviral response, and cell death (Al‐Shabany et al., 2016; Hong et al., 2018; Li et al., 2017; Sims et al., 2018). (Heer et al., 2020) reported that SARS‐CoV‐2 infection upregulates PARPs in human lung cell lines and that overexpression of PARP10 depresses cellular NAD^+^ in vitro. RNAseq analysis of the lung of a deceased patient also revealed dysregulated expression of several PARPs (Heer et al., 2020). (Raines, Cheung, et al., 2021) found higher urinary quinolinate‐to‐tryptophan ratio in patients with COVID‐19 who had acute kidney injury compared to healthy controls, and suggested that SARS‐CoV‐2 infection may be associated with a NAD^+^ biosynthetic defect. A few clinical trials aiming to boost NAD^+^ levels in patients with COVID‐19 have been published but none reported the levels of NAD^+^ or its metabolites (Altay et al., 2021; Raines, Ganatra, et al., 2021). Many review articles have discussed the role of NAD^+^ in innate immune response to viral infections and have postulated NAD^+^ depletion as a potential contributor to the severity of COVID‐19. (Block & Kuo, 2022; Novak, 2022; Zheng et al., 2022). However, no previous study has prospectively evaluated the levels of NAD^+^ and its major metabolites in well‐characterized patients with COVID‐19 and appropriately matched controls, and related these levels to indices of disease severity and other outcomes. This prospective observational study determined whether COVID‐19 in adults is associated with NAD^+^ depletion in peripheral blood mononuclear cells (PBMCs) and in whole blood compared to non‐infected adults, and whether NAD^+^ depletion and circulating levels of its metabolites are associated with the severity of the inflammatory response, disease severity, and hospital outcomes. Because hospitalized patients with COVID‐19 are typically older and have high prevalence of chronic diseases and both age and chronic diseases may independently affect NAD^+^ levels (Bhasin et al., 2023; Camacho‐Pereira et al., 2016; McReynolds et al., 2021), we included two groups of un‐infected controls: a group of age‐ and sex‐matched adults with comorbidities and a second group of middle‐aged and older adults without comorbidities. We also measured the circulating concentrations of major metabolites of NAD^+^ using rigorous LC–MS methods. We hypothesized that NAD^+^ depletion, reflected by the concentrations of NAD^+^ in the whole blood and in PBMCs and plasma concentrations of NAD^+^ metabolites, in adults with COVID‐19 is associated with the severity of the inflammatory response, disease severity, and hospital outcomes.

## 2 METHODS

### 2.1 Study population

Two groups of study participants were enrolled prospectively in this observational study: (1) 56 adults, 18 years or older, who were hospitalized with a confirmed diagnosis of SARS‐CoV‐2 infection, and (2) 31 community dwelling, adults who were matched for sex and decade of age, and who had one or more comorbid conditions, similar to those that are commonly observed in patients with COVID‐19. We also compared the hospitalized COVID‐19 patients with another group of 30 healthy, community‐dwelling, middle‐aged, and older adults, who had been studied previously in a randomized controlled trial (Bhasin et al., 2023).

#### 2.1.1 Patients with COVID‐19

Hospitalized patients with COVID‐19 who had participated in the Brigham Women's Hospital/Harvard Cohorts BioRepository were approached for inclusion in our prospective observational study using an IRB‐approved protocol. The participants were 18 years or older, had SARS‐CoV‐2 infection confirmed by an approved diagnostic test. The participants provided informed consent and authorization for the use of personal health information in accordance with Health Insurance Portability and Accountability Act (HIPAA). Potential participants were excluded if they were in the intensive care unit for severe illness, required mechanical ventilation at the time of screening, had a history of kidney transplantation, or were receiving or expected to receive hemodialysis or peritoneal dialysis at screening. Given the unprecedented severity of the COVID pandemic, many patients were in potentially life‐saving clinical trials. For this reason, participation in research studies of remdesivir or other antiviral drugs, monoclonal antibodies, convalescent plasma, and dexamethasone as a part of their clinical care was allowed. Patients in non‐COVID‐related interventional trials were excluded. The patients enrolled in placebo‐controlled intervention trials of other anti‐inflammatory or immunomodulatory agents were excluded. Occasional use of acetaminophen and nonsteroidal anti‐inflammatory drugs, such as ibuprofen, for fever or headache was permitted. Women who were pregnant or breastfeeding were excluded.

Hospital outcomes were ascertained from the data available in the electronic medical record. The participants were followed in the hospital for the duration of their hospital stay.

#### 2.1.2 The comparator group 1: Community‐dwelling, medically stable, age‐ and sex‐matched adults with comorbidities but without current SARS‐CoV‐2 infection

Patients hospitalized with COVID‐19 have high prevalence of chronic co‐morbid conditions that can independently affect NAD^+^ levels. Therefore, the control group enrolled non‐infected, adults, living within the greater Boston, MA area, 18 years or older, matched for sex and decade of age, who had similar co‐morbid conditions but were medically stable, did not have any acute illness and were willing and able to provide consent. The participants were excluded if they had: uncontrolled diabetes mellitus defined as hemoglobin A_1C_ >8.0%; hospitalization for an acute illness or major surgical procedure in the 3 months prior to screening; chronic kidney disease (serum creatinine >2 mg/dL); liver disease (ALT or AST >3 times the upper limit of normal); history of cancer other than nonmelanotic skin cancer, with treatment in the prior 2 years; untreated hypothyroidism or hyperthyroidism; or untreated and uncontrolled hypertension.

The participants in the control group were recruited by advertisement in the community and from other system‐based registries using IRB‐approved procedures. After obtaining informed consent, eligible participants underwent study assessments.

#### 2.1.3 Medically stable, healthy control population

We also included in the analyses a second group of 30 healthy, community‐dwelling, middle‐aged, and older adults (Pencina et al., 2023). Details of this population have been published (Pencina et al., 2023). Briefly, healthy community‐dwelling older men and postmenopausal women, 45 years or older, without significant health problems were recruited for a randomized, placebo‐controlled trial of β nicotinamide mononucleotide for 28 days. Baseline data prior to study drug administration were used to compare NAD^+^ and its metabolites, and inflammatory markers with hospitalized participants with the SARS‐CoV‐2 infection.

### 2.2 Measurements of NAD ^+^ and NAD ^+^ metabolites

To ensure pre‐analytical stability of the analytes, whole blood was collected from a fasting, morning (6–8 am) blood draw in 4% trichloroacetic acid and centrifuged, and supernatant was stored at −80°C. Detailed methods for measurements of NAD^+^ and NAD^+^ metabolites (nicotinamide, 1‐methylnicotinamide, and N‐methyl‐2‐pyridone‐5‐carboxamide [2‐PY]) have been published (Pencina et al., 2022, 2023). Briefly, blood NAD^+^ concentrations were measured using validated liquid chromatography tandem mass spectrometry method. The linear range for NAD 5.0–500.0 μg/mL. The inter‐assay coefficients of variation (CV) for the NAD+ assay were 6.0% and 8.7%, respectively, and intra‐assay CVs 8.9% and 9.1%, respectively.

### 2.3 Inflammatory markers

Serum specimens were collected after an 8‐h overnight fast during a morning visit and stored at −80°C until measurement. Serum interleukin 6 (IL‐6) was measured by a two‐site chemiluminescent immunoassay with intra‐assay and inter‐assay CVs 1.7%–4.6% and 3.1%–12.0%, respectively (Access Systems, Beckman Coulter, Inc. Fullerton, CA). Human TNF‐α was measured using an enzyme‐linked immunosorbent assay (ELISA) with intra‐ and inter‐assay CVs 3.1%–8.5% and 7.3%–10.6%, respectively (R & D Systems Inc., Minneapolis, MN). High sensitivity C‐reactive protein (CRP) was measured by a validated ELISA; intra‐assay and inter‐assay CVs were 3.8%–8.3% and 6.0%–7.0%, respectively (R & D Systems Inc., Minneapolis, MN).

### 2.4 RNA sequencing analyses

Blood for the isolation of PBMCs was collected in ethylenediamine tetraacetic acid (EDTA) tubes. Total RNA extracted from PBMCs of 11 participants with COVID‐19 and 8 participants from the age‐ and sex‐matched comparator group was subjected to RNA sequencing analysis. The mRNA sequencing via poly(A) selection preparation workflow included: (1) mRNA enrichment and fragmentation, and random priming, (2) first and second strand cDNA synthesis, (3) end repair, 5′ phosphorylation, and A‐tailing, followed by (4) adaptor ligation, polymerase chain reaction (PCR) enrichment, and sequencing.

The mRNA sequencing was performed by Azenta Life Sciences. Trimmomatic v.036 was used to remove potential adaptor sequences and nucleotides with poor quality (Bolger et al., 2014). Trimmed reads were mapped to ENSEMBL GRCh38 reference genome using the STAR aligner v.2.5.2b (Dobin et al., 2013). Reads that were unique and within exon regions were counted using Subread v.1.5.2 (Liao et al., 2014). Differential expression analysis was performed using DESeq2 (Love et al., 2014). *p*‐Values and log2 fold changes were generated using the Wald test. Differentially expressed genes (DEGs) were defined as genes with an absolute log2 fold change >2 or <0.5 and a *p*‐value < 0.05. The results were corrected using the Benjamini–Hochberg method. A gene set enrichment analysis (GSEA) was also performed using the R/Bioconductor implementation of FGSEA (Love et al., 2014).

RNA extracted from the PBMC samples from nine COVID‐19 participants and six participants from the comparator group were used for quantitative reverse transcription polymerase chain reaction (RT‐qPCR) analysis. RNA was reverse transcribed to complementary DNA, and RT‐qPCR was conducted in order to detect expression levels of target genes. Measured levels of the target genes were normalized to those of the housekeeping gene hypoxanthine phosphoribosyl transferase 1 (HPRT). Quality control was performed, and all samples had a DV200 value of ≥70% and an RNA integrity number ≥7. However, the cycle thresholds were too low or could not be determined in three COVID‐19 patients. Unpaired *t* tests were used to compare expression levels between the two groups.

### 2.5 Statistical methods

Baseline characteristics of the three analyzed populations were presented as numbers and percentages for categorical variables and mean, SD, median and IQR for continuous data. The pairwise comparisons between COVID‐19 patients and two non‐COVID‐19 cohorts were conducted using Mann–Whitney *U*‐ or chi‐squared tests. The distributions of NAD^+^ metabolites and inflammation factors were presented as box‐and‐whisker plots.

The association between the incidence of death (during approximately a 12‐month follow‐up) and escalation of care to intensive care unit (ICU) with NAD^+^ and its metabolites were performed using logistic regression. Odds ratios and corresponding 95% confidence intervals were extracted from these models. These analyses were repeated with adjustment for inflammatory markers (CRP, IL‐6, TNF‐α). Analyses of relationships between continuous hospital outcomes and NAD^+^ metabolites were performed using linear regression models. Sensitivity analyses were performed using log‐transformed data. Two‐sided type I error alpha was set at 0.05 for all hypotheses testing. Data analyses were conducted in SAS Version 9.4 (SAS Institute Inc., Cary, NC).

## 3 RESULTS

### 3.1 Study participants

Participant characteristics are summarized in Table 1. Briefly, participants hospitalized with COVID‐19 were middle‐aged or older (mean age 60.7 years, 95% CI: 57.2, 64.2), predominantly male (62.5%), White (85.7%), and overweight with a mean body mass index (BMI) 28.6 kg/m^2^, (95% CI: 26.8, 30.4). 20 (35.7%) participants were 65 years or older. The participants hospitalized with COVID‐19 had high prevalence of other co‐morbid conditions, notably, cardiovascular disease (57.1%), cancer (41.1%), and chronic lung disease (35.7%) than age‐ and sex‐matched controls.

**TABLE 1:** Characteristics of the prospectively enrolled study populations.

	COVID‐19 (*n* = 56)	Age‐ and sex‐matched non‐COVID‐19 control group (*n* = 31)	P (Covid −19 vs. age‐ and sex‐matched non‐COVID group)	Healthy control group (*n* = 30)	P (Covid vs. healthy control group)
Sex					
Female, *n* (%)	21 (37.5%)	13 (41.9%)	0.685	14 (46.7%)	0.410
Male, *n* (%)	35 (62.5%)	18 (58.1%)		16 (53.3%)	
Race *n* (%)					
Asian	1 (1.8%)	1 (3.3%)	0.342	0 (0%)	0.110
Black or African American	5 (8.9%)	6 (20.0%)		8 (26.7%)	
Other	2 (3.6%)	0 (0%)		0 (0%)	
White	48 (85.7%)	23 (76.7%)		22 (73.3%)	
More than One Race	0 (0%)	0 (0%)		0 (0%)	
Age (Years)	60.7 (13.0) 61.5 (53.0, 67.5)	59.1 (16.3) 59.0 (54.0, 70.0)	0.910	61.9 (8.6) 59.1 (56.9, 69.0)	0.720
Weight (kg)	86.2 (21.2) 85.8 (69.9, 96.2)	84.8 (17.6) 82.4 (74.4, 97.5)	0.989	85.5 (12.3) 83.5 (76.4, 95.1)	0.860
Height (cm)	173.5 (10.6) 172.7 (165.1, 180.3)	167.7 (7.2) 168.0 (164.0, 173.5)	0.014	171.1 (8.61) 172.0 (164.0, 176.6)	0.265
BMI (kg/m2)	28.59 (6.6) 28.7 (24.7, 31.8)	29.83 (5.4) 29.2 (26.6, 33.8)	0.189	29.2 (3.6) 27.9 (26.4, 32.0)	0.417
Medical conditions, *n* (%)					
Cancer history	23 (41.1%)	4 (12.9%)	0.007	—	—
Chronic kidney disease	8 (14.3%)	1 (3.3%)	0.105	—	—
Chronic liver disease	6 (10.7%)	1 (3.2%)	0.219	—	—
Chronic lung disease	20 (35.7%)	3 (9.7%)	0.008	—	—
Neurological conditions	10 (17.9%)	2 (6.5%)	0.140	—	—
Diabetes	14 (25.0%)	2 (6.5%)	0.033	—	—
Cardiovascular disease	32 (57.1%)	8 (25.8%)	0.005	—	—
Immunocompromised state	16 (28.6%)	1 (3.2%)	0.004	—	—
Mental health disorder	14 (25.0%)	3 (9.7%)	0.084	—	—
Transplant	5 (9.1%)	0 (0%)	0.084	—	—

*Note*: Table 1 Characteristics of the participants in the three study groups: patients hospitalized with COVID‐19; a prospectively enrolled control group of age‐ and sex‐matched adults with co‐morbidities; and a second control group of healthy middle‐aged and older adults. The first two groups—patients hospitalized with COVID‐19 and a control group of prospectively enrolled age‐ and sex‐matched adults with co‐morbidities were enrolled concurrently prospectively as a part of this study. The data on the second control group of healthy middle‐aged and older adults were derived from a previously published study and were included as a reference for comparison (Pencina et al., 2023).

### 3.2 Indices of disease severity

Indices of disease severity were obtained in the hospitalized patients with COVID‐19. The mean (95% CI) WHO ordinal disease severity score was 3.7 (3.5, 3.9), mSOFA scale score was 1.6 (1.1, 2.0), the length of hospital stay was 7.9 (6.2, 9.5) days, and the number of days with fever was 1.9 (1.3, 2.5). Three (5.4%) participants died during their hospitalization and an additional 10 (17.8%) died during the follow‐up period of up to 12 months for a total 1‐year mortality rate of 23.2%. Seven (12.5%) participants had worsening clinical condition requiring admission into the intensive care unit (ICU). Linear regression analyses did not show a significant relationship between NAD, NAM, Me‐NAM,2‐PY, 4‐PYR, and days of hospital stay or days of fever (Table S1).

### 3.3 NAD ^+^ and NAD ^+^ metabolites

**FIGURE 1:**
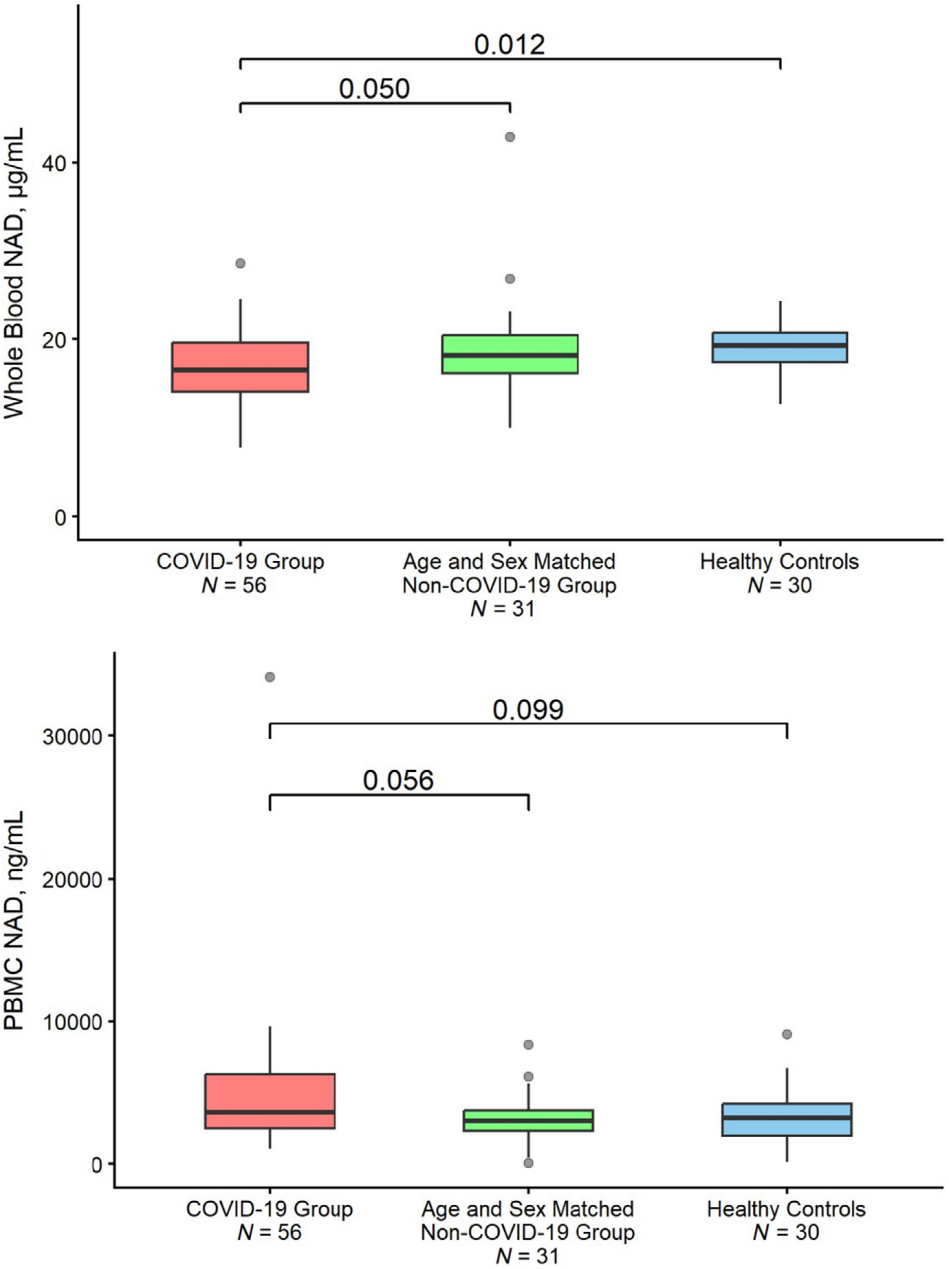
NAD^+^ levels in whole blood (Panel a) and PBMCs in the 3 groups: Three study groups patients hospitalized with COVID‐19; a control group of prospectively enrolled age‐ and sex‐matched adults with co‐morbidities; and a second control group of healthy middle‐aged and older adults. The first two groups—patients hospitalized with COVID‐19 and a control group of prospectively enrolled age‐ and sex‐matched adults with co‐morbidities were enrolled concurrently prospectively as a part of this study. The data on NAD^+^ levels in whole blood (Panel a) and PBMCs from a second control group of healthy middle‐aged and older adults were derived from a previously published study and were included as a reference for comparison (Pencina et al., 2023). The horizontal line in the box represents mean, the upper and lower margins of the box represent the 25th and 75th percentile values, and the whiskers represent the 95th percentile values.

The NAD^+^ concentrations in the whole blood and PBMCs are presented in Figure 1. Whole blood NAD^+^ levels were modestly but significantly lower in the patients infected with the SARS‐C0V‐2 infection compared to the control group of age‐ and sex‐matched adults with co‐morbidities (17.0 μg/mL, 95% CI: 15.9, 18.1 vs. 19.1 μg/mL, 95% CI: 16.9, 21.2; *p* = 0.05); The NAD^+^ levels in patients infected with the SARS‐C0V‐2 infection were also modestly lower than in healthy controls (17.0 μg/mL, 95% CI: 15.9, 18.1 vs. 18.9 μg/mL, 95% CI: 17.9, 20.0; *p* = 0.012). In those with COVID‐19, blood NAD^+^ levels did not differ significantly between patients less than 65 years and those 65 years or older (17.2 vs. 16.5 μg/mL; *p* = 0.473; Table S2). The NAD^+^ levels in the PBMCs were more variable and did not differ significantly among groups (5160.8 ng/mL, 95% CI: 3401.4, 6920.2 vs. 3228.0 ng/mL, 95% CI: 2558.9, 3897.1; *p* = 0.056).

**FIGURE 2:**
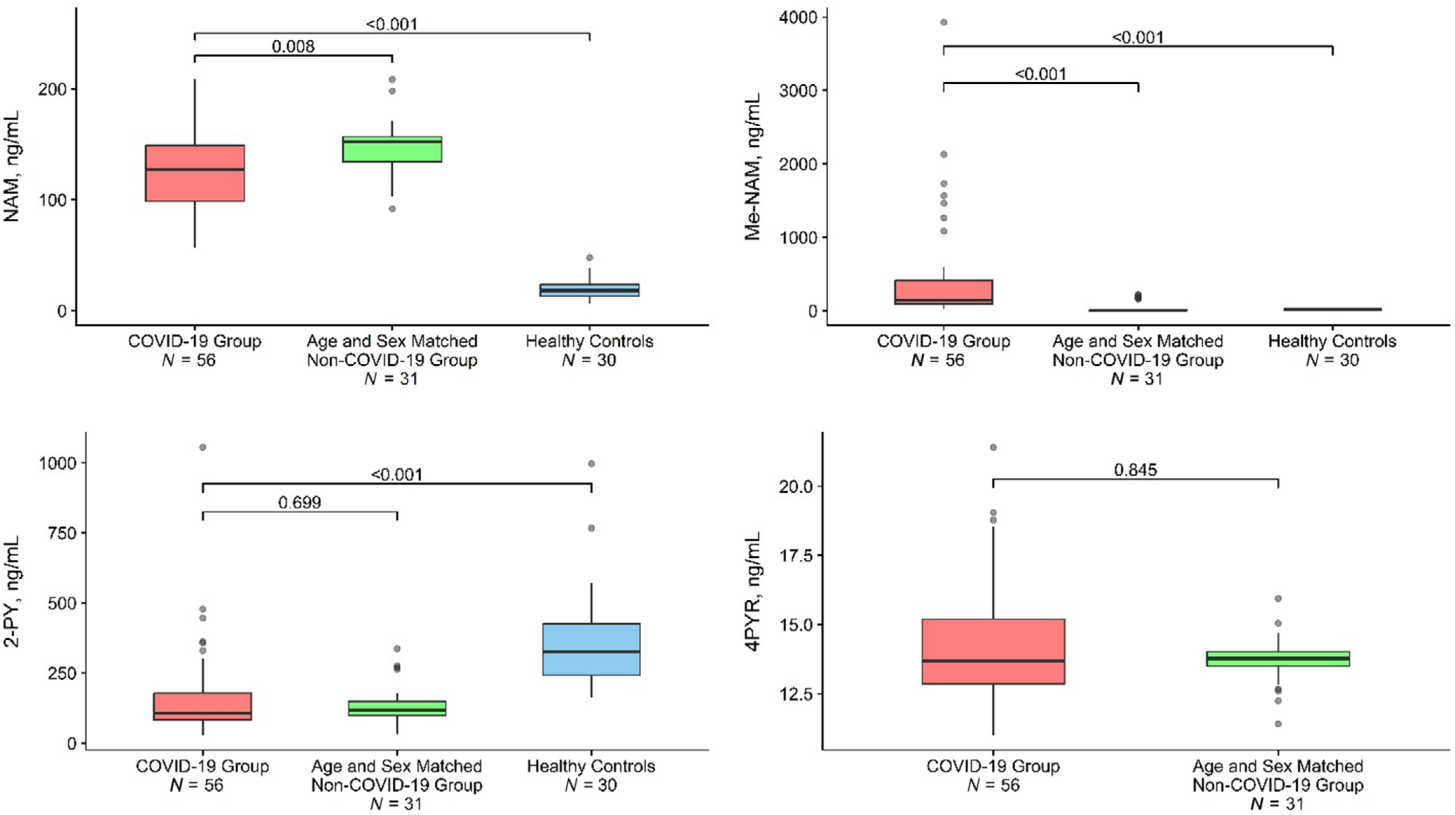
Circulating levels of NAD^+^ metabolites in COVID and in patients hospitalized with COVID‐19 and a control group of prospectively enrolled age‐ and sex‐matched adults with co‐morbidities. The horizontal line in the box represents mean, the upper and lower margins of the box represent the 25th and 75th percentile values, and the whiskers represent the 95th percentile values.

The plasma concentrations of major NAD^+^ metabolites are presented in Figure 2. The plasma concentrations of 1‐methylnicotinamide, a metabolite of NAD^+^ that cannot re‐enter the salvage pathway, were significantly higher in the COVID‐19 group (439.7 ng/mL, 95% CI: 234.0, 645.4) than in the age‐ and sex‐matched control group (44.5 ng/mL, 95% CI: 15.6, 73.4, *p* < 0.001) as well as the healthy controls (18.1 ng/mL, 95% CI 15.4, 20.8, *p* < 0.001). The circulating concentrations of nicotinamide, an NAD^+^ metabolite which can be recycled back to NAD^+^ through the salvage pathway, were significantly higher in the COVID‐19 group (126.9 ng/mL, 95% CI: 115.8, 138.1) as well as in the age‐ and sex‐matched adults with comorbidities (145.5 ng/mL, 95% CI: 136.0, 155.0) than in heathy control group (20.1 ng/mL, 95% CI: 16.7, 23.5, *p* < 0.001). Plasma 2‐PY concentrations were significantly lower in the COVID‐19 group compared to the healthy control group (166.9 ng/mL, 95% CI: 118.4, 215.5 vs. 369.2 ng/mL, 95% CI: 299.8, 438.6, *p* < 0.001) but did not differ from those in the age‐ and sex‐matched adults with comorbidities (136.6 ng/mL, 95% CI: 112.0, 161.2, *p* = 0.699). Plasma 4‐pyridone‐3‐carboxamide‐1‐β‐D‐ribonucleoside (4PYR) concentrations were similar in the COVID‐19 group and the age‐ and sex‐matched control group (14.4 ng/mL, 95% CI: 13.7, 15.0 vs. 13.7 ng/mL, 95% CI: 13.4, 14.0, *p* = 0.845). Plasma nicotinamide levels were higher in those infected with SARS‐CoV‐2 who were under 65 years old than in those who were 65 years or older (136.5 ng/mL vs. 111.0 ng/mL *p* = 0.025); however, the circulating levels of other NAD^+^ metabolites did not differ significantly between patients less than 65 years and those 65 years or older (Table S2). 4PYR concentrations could not be measured in healthy controls due to insufficient available sample.

### 3.4 The markers of inflammation

**FIGURE 3:**
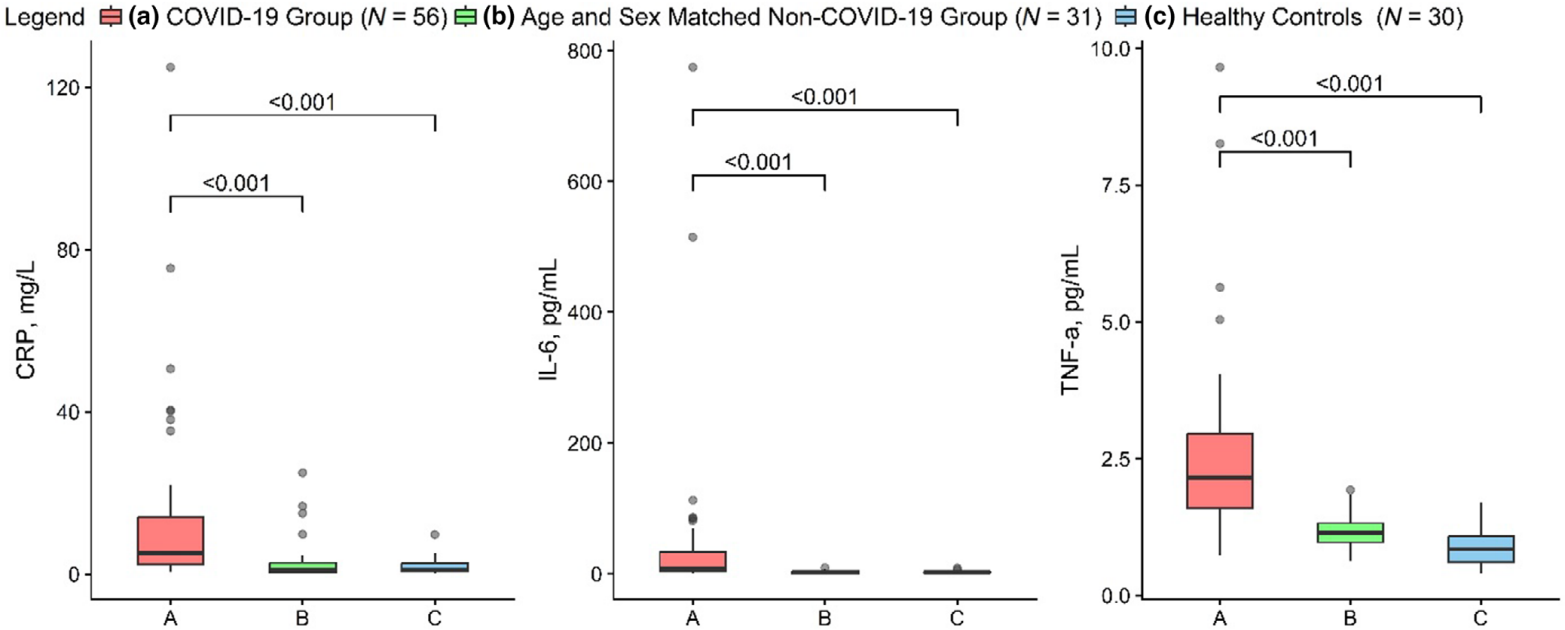
Circulating levels of inflammatory markers: CRP (left panel), IL‐6 (middle panel), and TNF‐alpha (right panel) in COVID‐19 group (a), age‐ and sex‐matched Non‐COVID‐19 group (b), and healthy controls (c).

Circulating concentrations of the markers of systemic Inflammation, CRP, TNF‐α, and IL‐6 (Figure 3) were substantially higher in the COVID‐19 group compared with age‐ and sex‐matched adults with co‐morbidities (CRP 13.0 mg/L, 95% CI: 7.3, 18.7 vs. 3.5 mg/L, 95% CI: 1.2, 5.7, *p* < 0.001; IL‐6 44.4 pg/mL, 95% CI: 10.5, 78.4 vs. 2.7 pg/mL, 95% CI: 1.9, 3.5, *p* < 0.001; and TNF‐α 2.5 pg/mL, 95% CI: 2.1, 3.0 vs. 1.2 pg/mL, 95% CI: 1.1, 1.3, *p* < 0.001). The circulating concentrations of CRP, TNF‐α, and IL‐6 in the COVID‐19 group as well as in the age‐ and sex‐matched adults with comorbidities were higher than those in the healthy controls (Figure 3).

### 3.5 Associations of NAD ^+^ and its metabolites with inflammatory markers and indices of disease severity

Neither the whole blood nor the PBMC NAD^+^ levels were associated significantly with any of the inflammation markers, mortality, risk of ICU transfer, or the length of stay. In contrast to NAD^+^, circulating levels of 2‐PY were significantly associated with the risk of death and worsening clinical status indicated by transfer to ICU (Table 2). A one standard deviation increase in the circulating 2‐PY level was associated with an increased risk of death (HR = 3.65, 95% CI: 1.09, 12.2, *p* = 0.036); this association was not attenuated by controlling for inflammatory factors CRP, IL‐6, or TNF‐α (HR = 3.62 95% CI: 1.00, 13.1, *p* = 0.049 after adjusting for these inflammatory markers). Higher 2‐PY levels were also associated with an increased risk of ICU admission (HR = 2.90, 95% CI: 1.01, 8.38, *p* = 0.049) which was not attenuated after adjusting for inflammatory factors (HR = 7.86, 95% CI: 1.07, 57.8, *p* = 0.043).

**TABLE 2:** Risk of poor hospital outcomes by standardized NAD^+^ and metabolite levels, adjusting for age and sex.

	Death OR (95% CI)	Death P	ICU or similar OR (95% CI)	ICU or similar P
PBMC NAD (Standardized)	0.63 (0.17, 2.32)	0.484	0.94 (0.36, 2.42)	0.893
PBMC NAD (Standardized) Mod 1 (CRP, IL‐6, TNF‐α)	0.32 (0.07, 1.44)	0.139	2.77 (0.21, 36.3)	0.437
Whole Blood NAD (Standardized)	0.77 (0.37, 1.63)	0.500	1.79 (0.70, 4.57)	0.226
Whole Blood NAD (Standardized) Mod 1 (CRP, IL‐6, TNF‐α)	0.91 (0.40, 2.04)	0.811	0.97 (0.24, 4.03)	0.970
NAM (Standardized)	0.56 (0.25, 1.24)	0.153	0.67 (0.25, 1.82)	0.435
NAM (Standardized) Mod 1 (CRP, IL‐6, TNF‐α)	0.44 (0.17, 1.15)	0.095	0.62 (0.15, 2.57)	0.514
Me‐NAM (Standardized)	1.23 (0.66, 2.28)	0.520	1.69 (0.76, 3.75)	0.196
Me‐NAM (Standardized) Mod 1 (CRP, IL‐6, TNF‐α)	1.44 (0.74, 2.80)	0.279	1.34 (0.51, 3.48)	0.551
2‐PY (Standardized)	3.65 (1.09, 12.2)	0.036	2.90 (1.01, 8.38)	0.049
2‐PY (Standardized) Mod 1 (CRP, IL‐6, TNF‐α)	3.62 (1.00, 13.1)	0.049	7.86 (1.07, 57.8)	0.043
4‐PYR (Standardized)	2.15 (0.99, 4.67)	0.053	1.56 (0.67, 3.63)	0.304
4‐PYR (Standardized) Mod 1 (CRP, IL‐6, TNF‐α)	2.23 (0.98, 5.07)	0.054	2.08 (0.69, 6.26)	0.191

### 3.6 RNA sequencing analyses

**FIGURE 4:**
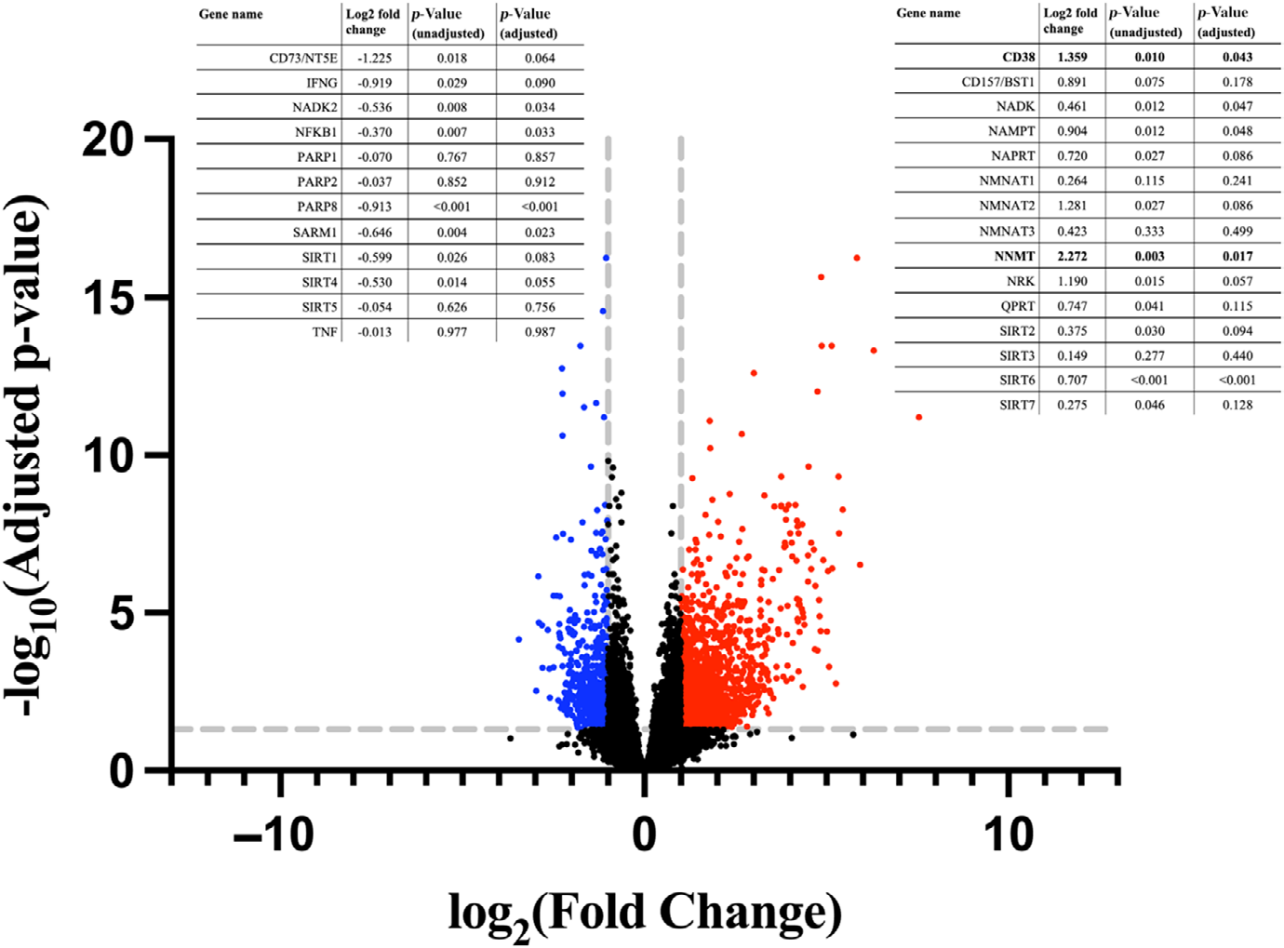
Volcano plot showing differentially expressed genes in the COVID‐19 group (*n* = 11) as compared to the matched control group (*n* = 8). Differentially expressed genes (DEGs) were defined as genes with an absolute log2 fold change >1 and an adjusted *p*‐value of <0.05. Down‐regulated DEGs are shown in blue and up‐regulated DEGs are in red, with select labeled genes in violet. Expression data from select genes is shown in overlying tables; genes meeting DEG criteria are listed in bold.

A comparison of expression profiles between the COVID‐19 and the age‐ and sex‐matched control group revealed 2145 genes meeting predefined differentially expressed gene (DEG) criteria (an arbitrary cut‐off for a fold change of >2 or <0.5; adjusted *p* < 0.05): 1601 genes were upregulated in the COVID‐19 group and 544 genes were downregulated (Figure 4 and Table 3). Differentially expressed genes encoded for proteins involved in NAD^+^ metabolism (e.g., NNMT: upregulated 4.8‐fold in the COVID‐19 group) and the immune response (e.g., IL17B: upregulated 2.8‐fold), as well as proteins known to deplete NAD^+^ (e.g., CD38: upregulated 2.6‐fold). Of note, numerous additional genes in each of these three domains showed some degree of upregulation or downregulation in the COVID‐19 group but did not meet formal DEG criteria due to a fold‐change value of insufficient magnitude or a *p*‐value that became non‐significant after statistical adjustment (select genes are listed in Table 3, genes involved in NAD synthesis are presented in Table S3). Furthermore, GSEA revealed differential expression of numerous pathways which utilize NAD^+^ in the COVID‐19 group, including those involved in immune function, DNA repair, carbohydrate and fat metabolism, apoptosis/autophagy, redox reactions, and mitochondrial function (Table 3).

**TABLE 3:** Significant (*p* < 0.05) pathways of interest as identified by gene set enrichment analysis (GSEA).

Pathway	ES	NES	Size	*p*‐Value
Immune System/Inflammation				
Activation of NF‐kB in B cells	0.563	1.732	66	<0.001
Chemokine receptors bind chemokines	−0.485	−1.638	44	0.005
ROS and RNS production in phagocytes	0.688	1.824	29	<0.001
Signaling by interleukins	0.372	1.351	394	0.003
DNA Damage Response/DNA Repair				
Base excision repair	0.619	1.939	75	<0.001
Mismatch repair	0.686	1.602	15	0.011
p53‐dependent G1 DNA damage response	0.640	1.963	65	<0.001
p53‐independent DNA damage response	0.699	2.045	51	<0.001
Carbohydrate/fat metabolism				
Metabolism of carbohydrates	0.397	1.400	246	0.008
The citric acid TCA cycle and respiratory electron transport	0.645	2.207	169	<0.001
Apoptosis/autophagy				
Autophagy	0.460	1.554	139	0.001
Regulation of apoptosis	0.687	2.026	52	<0.001
Mitochondrial function				
Mitochondrial biogenesis	0.453	1.446	94	0.011
Mitochondrial protein import	0.567	1.709	61	0.001
Mitochondrial translation	0.657	2.094	93	<0.001

*Note*: “Size” is the number of genes in the given pathway.

Abbreviations: ES, Enrichment score; NES, Normalized enrichment score.

### 3.7 mRNA quantitation using reverse transcription‐quantitative polymerase chain reaction (RT‐qPCR)

**FIGURE 5:**
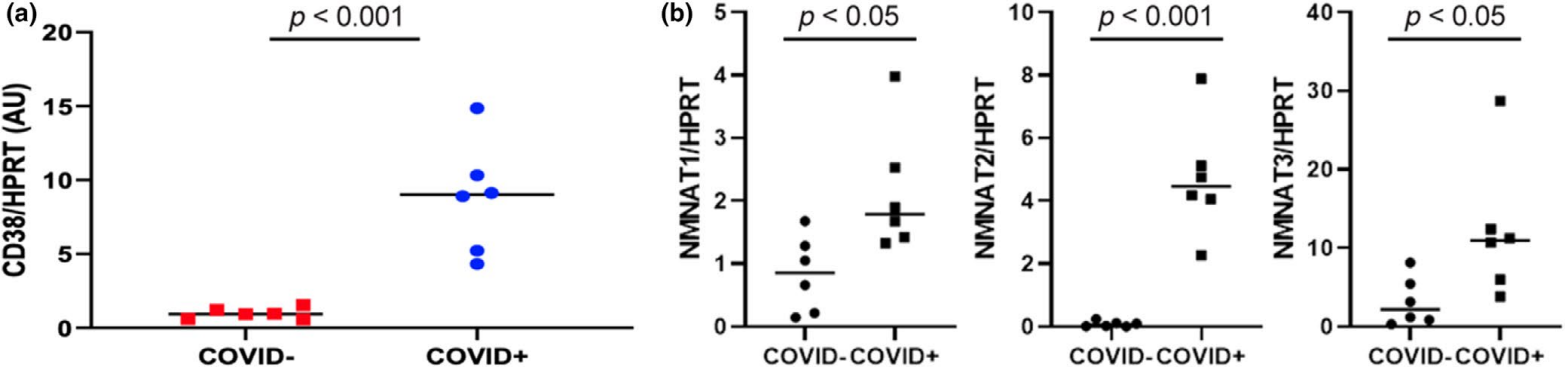
Quantitative reverse transcription polymerase chain reaction (RT‐qPCR) analysis comparing RNA levels of target genes in the COVID‐19 (*n* = 6) and matched control (*n* = 6) groups. Target genes were CD38 (a), and the nicotinamide mononucleotide adenylyl transferases (b). The hypoxanthine phosphoribosyl transferase 1 (HPRT) gene was used for normalization. Median values for each group are shown (represented by horizontal lines).

Due to limited amount of RNA available, we performed RT‐qPCR analyses of RNA isolated from PBMCs to determine the expression levels of some of the enzymes involved in NAD^+^ biosynthesis and degradation (Figure 5). The mRNA expression levels of CD38, a major NAD^+^ degrading enzyme (level normalized to HPRT: 8.80 vs. 0.97; *p* < 0.001), and nicotinamide mononucleotide adenylyl transferase 1 (NMNAT1; 2.18 vs. 0.85; *p* = 0.018), nicotinamide mononucleotide adenylyl transferase 2 (NMNAT2; 4.68 vs 0.09; *p* < 0.001), and nicotinamide mononucleotide adenylyl transferase 3 (NMNAT3; 12.01 vs. 2.81; *p* = 0.032) were all substantially higher in the COVID‐19 group compared to age‐ and sex‐matched controls.

## 4 DISCUSSION

To our knowledge, this is the first prospective observational study to systematically evaluate NAD^+^ and its metabolites in patients hospitalized with COVID‐19. In patients with moderately severe COVID‐19, whole blood, steady‐state NAD^+^ concentrations were only modestly lower compared to an age‐ and sex‐matched control group with comorbidities, as well as a healthy control group without comorbidities. In contrast, the circulating concentrations of the NAD^+^ metabolite, 1‐methylnicotinamide, were significantly higher in the COVID‐19 group than in both control groups. Circulating nicotinamide concentrations were also higher in COVID‐19 group compared with the healthy control group. RNAseq and mRNA expression levels revealed marked upregulation of multiple genes involved in NAD^+^ biosynthesis as well as of several enzymes involved in NAD^+^ consumption and degradation. CD38, an NAD^+^‐dependent ectoenzyme that is implicated in inflammation and NAD^+^ degradation and has been hypothesized to be a link between NAD^+^ metabolism and inflammatory response in COVID‐19 (Zeidler et al., 2022) was markedly upregulated in both RT‐qPCR and RNAseq analyses of COVID‐19 subjects as were NMNAT isoforms and NAMPT, both important enzymes in the NAD^+^ salvage pathway (Garten et al., 2015). The substantially higher concentrations of NAD^+^ metabolites—1‐methylnicotinamide and nicotinamide—in patients with COVID‐19 provide corroboration of the increased NAD^+^ turnover and reduced efficiency at recycling NAM, possibly due to limited availability of PRPP for nucleotide synthesis (Migaud et al., 2020; Perla‐Kajan & Jakubowski, 2022).

These alterations in NAD^+^ and its metabolome and increased turnover were accompanied by dysregulation of a number of downstream cellular pathways in which NAD^+^ serves as a co‐factor. Over 2000 genes met formal DEG criteria in RNA sequencing analysis. DEGs included those involved in immune function (e.g., IL17B, IL2RB) as well as those encoding for enzymes known to deplete NAD^+^ (e.g., CD38). The expression levels of several gene products involved in inflammation and immune response were dysregulated consistent with the observed increase in the circulating levels of downstream inflammatory markers TNF‐α, IL‐6, and CRP. Furthermore, gene set analysis revealed dysregulation in additional NAD^+^‐dependent pathways, such as DNA repair, redox reactions, epigenetic regulation, and mitochondrial function. These expression analyses provide additional supportive evidence of increased NAD^+^ turnover and dysregulation of downstream NAD^+^‐dependent processes in patients with COVID‐19.

NAD^+^ depletion has been proposed as a mechanism of disease severity in COVID‐19 and other viral illnesses (Radenkovic & Verdin, 2020). In vitro studies evaluating NAD^+^ metabolism in human Zika Virus infection (Sahoo et al., 2023) and murine hepatitis virus infection have shown altered NAD^+^ metabolism (Heer et al., 2020). Similarly, RNA sequencing of lungs from transgenic mice expressing human ACE2 revealed that SARS‐CoV‐2 infection upregulates molecules involved in both NAD synthesis and degradation (Izadpanah et al., 2023). Analysis of a lung biopsy from a single individual who died of COVID‐19 infection suggested downregulated synthesis of NAD^+^ from tryptophan and nicotinic acid (NA) (Heer et al., 2020). SARS‐CoV‐2 infection was shown to upregulate MARylating PARPs that can depress cellular NAD^+^ (Perla‐Kajan & Jakubowski, 2022). Metabolomic profiling in serum of patients with COVID‐19 found that NAD^+^ was reduced in more severe cases of COVID‐19 (Xiao et al., 2021) but the measurements of NAD^+^ in the serum may not be meaningful. However, in our systematic study of a much larger sample of hospitalized patients with COVID‐19, whole blood NAD^+^ was only slightly decreased relative to both control groups (Pencina et al., 2022). Furthermore, the blood NAD^+^ levels were not associated with risk of death, escalation to ICU stay, or circulating levels of inflammation markers.

The clinical significance of upregulation of nicotinamide N‐methyl transferase and the elevated circulating N‐methylnicotinamide levels in patients with COVID‐19 needs further investigation. Increased levels of N‐methylnicotinamide have been reported to exert vasoprotective, anti‐inflammatory, and anti‐thrombotic roles and improve endothelial function in some physiologic states (Bar et al., 2017; Campagna et al., 2021; Nejabati et al., 2018). However, an increase in NNMT expression and methylated catabolites of nicotinamide has also been reported to contribute to renal fibrosis (Takahashi et al., 2022) and fatty liver (Komatsu et al., 2018) in other experimental models.

The circulating levels of 2‐PY, a derivative of 1‐methylnicotinamide, were significantly associated with the risk of death and worsening clinical status reflected in the escalation of care by transfer to the ICU. 2‐PY is a major NAD^+^ metabolite that increases with age and illness (Slominska et al., 2004) and has been recognized as a uremic toxin in patients with kidney disease (Lenglet et al., 2016) that may exert toxic effects on the tissues (Rutkowski et al., 2003) and inhibit PARP‐1 activity (Slominska et al., 2006). The potential application of circulating 2‐PY level as a biomarker of adverse outcomes in patients hospitalized with COVID‐19 needs further investigation.

Overall, we did not find that NAD+ depletion was a contributor to the severity or outcomes of COVID‐19 or major age‐related differences in the levels of NAD+, as has been reported previously (Block & Kuo, 2022; Breton et al., 2020; Clement et al., 2019; Novak, 2022; Zheng et al., 2022). It has generally been assumed that NAD^+^ concentrations in the blood are markedly depleted in acute illnesses such as that associated with SARS‐CoV‐2 infection (Heer et al., 2020; Izadpanah et al., 2023; Sahoo et al., 2023). Our data in patients with SARS‐CoV‐2 infection show that blood NAD^+^ concentrations are only modestly lower but that the circulating concentrations of NAD^+^ metabolites are increased substantially suggesting increased turnover. These findings have clinical and physiologic implications. First, the concept that NAD^+^ is greatly depleted during acute SARS‐CoV‐2 infections seems simplistic; instead, modest reductions in blood NAD^+^ levels are accompanied by increased turnover. McReynolds et al. also found that the decline in NAD^+^ with normal aging was relatively subtle and was associated with increased turnover (Pencina et al., 2023). Second, in light of the increased turnover during the acute SARS‐CoV‐2 infection, it is possible that higher doses of NAD^+^ precursors may be needed to increase NAD^+^ levels in people with acute SARS‐CoV‐2 infection. Third, the concentrations of some NAD^+^ metabolites are markedly increased in SARS‐CoV‐2 infection and some of these metabolites could independently affect disease outcomes. A recent study reported that the NAD+ metabolite 2PY is associated with major cardiovascular‐associated events (Ferrell et al., 2024); in our study, 2PY was associated with mortality and increase in the level of hospital care in those infected with COVID‐19. While we did not evaluate cardiovascular outcomes in our study, we found that participants in the COVID‐19 group who had pre‐existing cardiovascular disease had higher 4‐PYR in unadjusted analyses (Table S4). Randomized controlled studies are needed to evaluate the safety and ability of NAD^+^ precursors to raise NAD^+^ levels in host tissues including blood.

The findings of this observational study should be viewed in the context of its strengths and limitations. This is the first study to prospectively evaluate the concentrations of NAD^+^ and its metabolites in COVID‐19 patients and relate them to hospital outcomes. The study included a prospectively enrolled, age‐ and sex‐matched control group with co‐morbid conditions similar to those that are prevalent in COVID‐19 patients; in addition, we compared the levels of NAD^+^ and its metabolites to those in healthy, middle‐aged, and older adults. The samples were collected using a standardized protocol to ensure pre‐analytical stability, and NAD^+^ and its metabolites were measured using validated LC–MS methods. Even though this is the largest study of NAD^+^ and its metabolites in COVID‐19 patients, the sample size was relatively small which may have limited the statistical power to detect significant associations. The confidence intervals and the *p*‐values were not adjusted for multiplicity; therefore, the *p*‐values from the association analyses should be considered nominal. By intent, patients who required admission to an intensive care unit, mechanical ventilation, or hemodialysis at screening were excluded. Similarly, patients with mild COVID‐19 disease who did not require hospitalization were excluded. Although sample collection in trichloroacetic acid stabilized NAD^+^ and its metabolites, this procedure precluded measurements of NADH, NADP, and NADPH. NAD^+^ has been reported to be present in circulating blood cells other than PBMCs (e.g., red blood cells and platelets) whose counts may vary during COVID‐19 and which were not studied.

## 5 CONCLUSIONS

In hospitalized patients with COVID‐19, NAD^+^ turnover is increased in and in spite of upregulation of enzymes involved in NAD^+^ biosynthesis, the steady‐state NAD+ levels are modestly reduced due to marked increase in the expression levels of NAD^+^ consuming/degrading enzymes resulting in dysregulation of a number of downstream cellular pathways in which NAD^+^ serves as a co‐factor. Further studies are needed to investigate the mechanisms by which SARS‐CoV‐2 increases NAD+ turnover and to test the hypothesis that NAD^+^ augmentation in patients with COVID‐19 is safe and can attenuate the severity of the dysregulated inflammatory response and multi‐organ injury associated with the infection.

## AUTHOR CONTRIBUTIONS

RV contributed to study design, study conduct, data analysis, data interpretation, manuscript preparation and revision. SB contributed to study conduct, data interpretation, manuscript preparation and revisions. BW contributed to study design, data analysis and manuscript preparation. YS and KP contributed to data analysis. LW, AB, YM and LP contributed to study conduct, and critical revisions of the manuscript. DJL, MMnt, PS, PL, SL, MMig, JC, NL contributed to data interpretation and critical revisions of the manuscript.

## FUNDING INFORMATION

This study was funded by Metro International Biotech, LLC. Drs. Rodrigo J Valderrábano and Shalender Bhasin were supported in part by Boston Claude D. Pepper Older Americans Independence Center grant P30AG031679. We are grateful to the clinicians and phlebotomists who enabled access to hospitalized patients and obtained blood samples without which this study would not have been possible.

## CONFLICT OF INTEREST STATEMENT

Dr. Bhasin's institution has received grants from NIA, NICHD‐NCMRR, DoD, AbbVie, Besins, FPT, and Metro International Biotech, on which Dr. Bhasin serves as the principal investigator, and consulting fees from Besins, Novartis, and Varsenis. These conflicts are overseen by the Office of Industry Interaction of Mass General Brigham in accordance with institutional rules and regulations. DJL, PS, PL, and SL are employed by Metro International Biotech. MMnt receives support from the Merck Investigators Studies Program and the Claude D. Pepper OAIC. MMnt is also the Editor‐in‐Chief of Aging Cell and was blinded to the review of this manuscript. RV, BW, KP, YS, NL, MMig, JC, LW, LP, YM, and AB have no conflicts of interest to declare.

## Supporting information


Table S1.

Table S2.

Table S3.

Table S4.

## Data Availability

The data that support the findings of this study are available from the corresponding author upon reasonable request.
