# Supplementary material for: Dysregulated nicotinamide adenine dinucleotide metabolome in patients hospitalized with COVID‐19
Source: Aging Cell. 2024 Oct 1;23(12):e14326. doi: 10.1111/acel.14326 (PMC11634700; doi:10.1111/acel.14326)
Supplement: Supplementary file 1 — Table S1. [file ACEL-23-e14326-s003.docx]

|  | **Days of Hospital Stay B (95% CI)** | **Hospital Stay P** | **Days of Fever B (95% CI)** | **Fever P** |
| --- | --- | --- | --- | --- |
| PBMC NAD (Standardized) | 0.13 (-1.9, 2.18) | 0.897 | 0.08 (-.64, 0.80) | 0.826 |
| PBMC NAD (Standardized) Mod 1 (CRP, IL-6, TNF-a) | 0.10 (-2.6, 2.77) | 0.942 | -.21 (-1.2, 0.76) | 0.663 |
| Whole Blood NAD (Standardized) | 0.53 (-1.4, 2.42) | 0.572 | -.31 (-.97, 0.36) | 0.361 |
| Whole Blood NAD (Standardized) Mod 1 (CRP, IL-6, TNF-a) | 0.15 (-1.8, 2.06) | 0.872 | -.45 (-1.2, 0.26) | 0.205 |
| NAM (Standardized) | 0.58 (-1.4, 2.53) | 0.550 | 0.35 (-.32, 1.02) | 0.293 |
| NAM (Standardized) Mod 1 (CRP, IL-6, TNF-a) | 0.32 (-1.5, 2.12) | 0.717 | 0.23 (-.44, 0.91) | 0.492 |
| Me-NAM (Standardized) | -.12 (-2.2, 1.95) | 0.910 | -.13 (-.85, 0.58) | 0.709 |
| Me-NAM (Standardized) Mod 1 (CRP, IL-6, TNF-a) | 0.07 (-1.8, 1.99) | 0.942 | -.01 (-.74, 0.71) | 0.972 |
| Me-NAM (Standardized) | -.12 (-2.2, 1.95) | 0.910 | -.13 (-.85, 0.58) | 0.709 |
| Me-NAM (Standardized) Mod 1 (CRP, IL-6, TNF-a) | 0.07 (-1.8, 1.99) | 0.942 | -.01 (-.74, 0.71) | 0.972 |
| 2-PY (Standardized) | 0.21 (-1.8, 2.19) | 0.831 | -.28 (-.97, 0.40) | 0.409 |
| 2-PY (Standardized) Mod 1 (CRP, IL-6, TNF-a) | -.02 (-1.8, 1.78) | 0.978 | -.32 (-.99, 0.35) | 0.342 |
| 4-PYR (Standardized) | -1.1 (-3.0, 0.85) | 0.267 | -.51 (-1.2, 0.15) | 0.127 |
| 4-PYR (Standardized) Mod 1 (CRP, IL-6, TNF-a) | -1.1 (-2.9, 0.61) | 0.196 | -.46 (-1.1, 0.19) | 0.156 |
|  | | | | |
| NAD: Nicotinamide adenine dinucleotide NAM: nicotinamide Me-NAM: 1-methylnicotinamide 2-PY: 2-methyl-2-pyridone-5-carboxamide 4-PYR: 4-pyridone-3-carboxamide-1-β-D-ribonucleoside | | | | |
