# Supplementary material for: Dysregulated nicotinamide adenine dinucleotide metabolome in patients hospitalized with COVID‐19
Source: Aging Cell. 2024 Oct 1;23(12):e14326. doi: 10.1111/acel.14326 (PMC11634700; doi:10.1111/acel.14326)
Supplement: Supplementary file 2 — Table S2. [file ACEL-23-e14326-s002.docx]

|  | **SARS-CoV-2 (n=56)** | | | **Matched Non-SARS-CoV-2 (n=31)** | | | | **Completely Healthy Group (MIB 28 Day) (n=30)** | | | |
| --- | --- | --- | --- | --- | --- | --- | --- | --- | --- | --- | --- |
|  | **Age<65 (n=36)** | **Age>=65 (n=20)** | **P** | **Age<65 (n=19)** | **Age>=65 (n=12)** | **P** | | **Age<65 (n=20)** | **Age>=65 (n=10)** | **P** | |
| **NAD** |  |  |  |  |  | |  |  |  | |  |
| Whole Blood NAD (µg/mL) | 17.2 (4.3)  16.5 (14.8, 19.9) | 16.5 (3.8)  16.2 (13.4, 19.3) | 0.473 | 18.6 (6.9)  17.4 (15.9, 18.9) | 19.8 (3.4)  20.2 (16.8, 22.1) | | 0.219 | 19.5 (2.8)  20.1 (17.6, 21.1) | 17.8 (2.2)  18.1 (15.6, 19.3) | | 0.095 |
| PBMC NAD (ng/mL) | 5744.4 (6332.1)  3880.0 (2920.0, 5760.0) | 4038.5 (2465.9)  3240.0 (2230.0, 6550.0) | 0.268 | 3407.6 (1962.6)  3120.0 (2040.0, 4550.0) | 2886.8 (1313.5)  2755.0 (2410.0, 3500.0) | | 0.506 | 3573.5 (1969.7)  3195.0 (2040.0, 4280.0) | 3085.3 (1694.8)  3345.0 (1660.0, 4280.0) | | 0.843 |
| **NAD-Related Metabolites** |  |  |  |  |  | |  |  |  | |  |
| NAM (ng/mL) | 136.5 (38.9)  135.0 (105.2, 158.7) | 111.0 (32.5)  107.7 (84.6, 128.2) | 0.025 | 140.5 (24.3)  142.2 (116.0, 153.8) | 153.5 (27.3)  154.9 (145.7, 165.7) | | 0.123 | 21.2 (10.0)  20.8 (13.2, 26.3) | 18.1 (7.0)  16.3 (12.8, 23.3) | | 0.441 |
| Me-NAM (ng/mL) | 322.5 (359.3)  157.4 (96.5, 408.0) | 635.0 (1050.0)  128.8 (88.7, 592.1) | 0.678 | 50.8 (82.4)  3.0 (1.1, 157.6) | 34.5 (75.2)  2.4 (1.3, 5.4) | | 0.919 | 19.4 (7.0)  16.6 (14.2, 26.8) | 15.7 (7.1)  16.0 (8.8, 22.0) | | 0.302 |
| 2-PY (ng/mL) | 179.8 (198.3)  108.7 (90.3, 177.7) | 145.6 (97.8)  100.3 (81.3, 180.6) | 0.647 | 136.5 (75.1)  116.4 (91.1, 147.2) | 136.8 (55.3)  127.0 (103.1, 158.5) | | 0.530 | 333.1 (146.1)  310.0 (220.0, 403.0) | 437.9 (230.0)  371.5 (251.0, 539.0) | | 0.169 |
| 4-PYR (ng/mL) | 14.1 (2.3) 13.4 (12.6, 14.8) | 14.8 (2.4) 14.0 (13.5, 16.1) | 0.190 | 13.6 (1.0) 13.8 (13.2, 14.1) | 13.8 (0.7) 13.8 (13.5, 14.0) | | 0.887 | - | - | |  |
| Values are expressed as mean (Standard Deviation) in the upper row and median (IQR) in the lower row.  P-values are calculated using the Mann-Whitney model.  NAD: Nicotinamide adenine dinucleotide NAM: nicotinamide Me-NAM: 1-methylnicotinamide 2-PY: 2-methyl-2-pyridone-5-carboxamide 4-PYR: 4-pyridone-3-carboxamide-1-β-D-ribonucleoside | | | | | | | | | | | |
