# Supplementary material for: Dysregulated nicotinamide adenine dinucleotide metabolome in patients hospitalized with COVID‐19
Source: Aging Cell. 2024 Oct 1;23(12):e14326. doi: 10.1111/acel.14326 (PMC11634700; doi:10.1111/acel.14326)
Supplement: Supplementary file 3 — Table S3. [file ACEL-23-e14326-s001.docx]

**Supplemental Table 3. Changes in the Expression levels of Genes Involved NAD Synthesis or Related genes: Results of the RNA Sequencing Analysis**

| **Gene name** | **Log2 fold change** | **p-value**  **(unadjusted)** | **p-value**  **(adjusted)** | **Description** |
| --- | --- | --- | --- | --- |
| **Salvage pathway** |  |  |  |  |
| NAMPT | 0.904 | 0.012 | 0.048 | Nicotinamide phosphoribosyltransferase |
| NMNAT1 | 0.264 | 0.115 | 0.241 | Nicotinamide nucleotide adenylyltransferase 1 |
| NMNAT2 | 1.281 | 0.027 | 0.086 | Nicotinamide nucleotide adenylyltransferase 2 |
| NMNAT3 | 0.423 | 0.333 | 0.499 | Nicotinamide nucleotide adenylyltransferase 3 |
| NMRK1 | -0.228 | 0.295 | 0.460 | Nicotinamide riboside kinase 1 |
| **Preiss-Handler Pathway** |  |  |  |  |
| NAPRT | 0.720 | 0.027 | 0.086 | Nicotinate phosphoribosyltransferase |
| **De novo synthesis** |  |  |  |  |
| 3-HAO | 0.413 | 0.276 | 0.440 | 3-hydroxyanthranilate 3,4-dioxygenase |
| AFMID | 0.287 | 0.173 | 0.319 | Arylformamidase |
| ASPDH | 0.089 | 0.826 | 0.896 | Aspartate dehydrogenase domain containing protein |
| IDO | 0.196 | 0.763 | 0.854 | Indoleamine 2,3-dioxygenase 1 |
| KMO | 0.344 | 0.377 | 0.542 | Kynurenine 3-monooxygenase |
| KYNU | 0.210 | 0.401 | 0.565 | Kynureninase |
| QPRT | 0.747 | 0.041 | 0.115 | Quinolinate phosphoribosyltransferase |
| SLC36A4 | 0.208 | 0.475 | 0.631 | Solute carrier family 36 member 4 |
| SLC7A5 | 0.712 | 0.073 | 0.175 | Solute carrier family 7 member 5 |
| **Other related genes** |  |  |  |  |
| NNMT | 2.272 | 0.003 | 0.017 | Nicotinamide N-methyltransferase |
| NADK | 0.461 | 0.012 | 0.047 | NAD kinase |
| NADK2 | -0.536 | 0.008 | 0.034 | NAD kinase 2 |
| PTGIS | 1.193 | 0.001 | 0.005 | Prostaglandin I2 synthase |
| LDHB | -0.065 | 0.824 | 0.895 | Lactate dehydrogenase B |
| NADSYN1 | 0.149 | 0.177 | 0.324 | NAD synthetase 1 |
| NNT | -0.421 | 0.003 | 0.018 | Nicotinamide nucleotide transhydrogenase |
| NAXD | -0.222 | 0.089 | 0.201 | NAD(P)HX dehydratase |
| SLC12A8 | 0.575 | 0.200 | 0.353 | Solute carrier family 12 member 8 |
| SLC25A51 | -0.259 | 0.114 | 0.240 | Solute carrier family 25 member 51 |
| PNP | 0.057 | 0.817 | 0.890 | Purine nucleoside phosphorylase |
| NUDT12 | -0.444 | 0.191 | 0.342 | Nudix hydrolase 12 |

Legend: Genes meeting differentially expressed gene criteria (absolute log2 fold change > 1, adjusted p-value of < 0.05) are listed in bold. Expression data was not available for certain genes including AOX1, IDO2, NMRK2, SLC22A13, SLC5A8, TDO.
