# Supplementary material for: Dysregulated nicotinamide adenine dinucleotide metabolome in patients hospitalized with COVID‐19
Source: Aging Cell. 2024 Oct 1;23(12):e14326. doi: 10.1111/acel.14326 (PMC11634700; doi:10.1111/acel.14326)
Supplement: Supplementary file 4 — Table S4. [file ACEL-23-e14326-s004.docx]

|  | **SARS-CoV-2 (n=56)** | | | **Matched Non-SARS-CoV-2 (n=31)** | | |
| --- | --- | --- | --- | --- | --- | --- |
|  | **No CVD (n=24)** | **Yes CVD (n=32)** | **P** | **No CVD (n=23)** | **Yes CVD (n=8)** | **P** |
| **NAD** |  |  |  |  |  |  |
| Whole Blood NAD (µg/mL) | 16.9 (4.0)  17.1 (14.9, 19.7) | 17.0 (4.2)  15.5 (13.6, 19.9) | 0.722 | 19.4 (6.4)  18.4 (16.2, 21.7) | 18.0 (2.6)  16.9 (15.9, 20.2) | 0.462 |
| PBMC NAD (ng/mL) | 4813.3 (2600.5)  4820.0 (2550.0, 6550.0) | 5387.4 (6614.4)  3380.0 (2370.0, 6470.0) | 0.492 | 3187.8 (1768.6)  3060.0 (2420.0, 3800.0) | 3354.3 (1862.0)  2360.0 (2040.0, 5660.0) | 0.858 |
| **NAD-Related Metabolites** |  |  |  |  |  |  |
| NAM (ng/mL) | 124.3 (40.4)  124.7 (96.0, 138.6) | 128.9 (37.5)  128.7 (100.5, 153.2) | 0.638 | 145.6 (27.8)  150.8 (128.9, 159.9) | 145.5 (21.2)  153.8 (135.4, 156.0) | 0.787 |
| Me-NAM (ng/mL) | 314.5 (410.9)  134.7 (95.5, 372.0) | 529.1 (857.2)  164.9 (93.5, 459.0) | 0.497 | 32.3 (64.4)  3.0 (1.2, 8.8) | 79.4 (108.2)  1.7 (0.9, 204.0) | 0.910 |
| 2-PY (ng/mL) | 157.3 (215.1)  98.8 (84.7, 138.1) | 173.8 (126.7)  118.3 (82.5, 243.8) | 0.282 | 138.2 (69.3)  122.7 (98.5, 150.6) | 132.2 (64.6)  115.2 (99.4, 152.4) | 0.910 |
| 4-PYR (ng/mL) | 13.5 (1.6)  13.1 (12.5, 14.0) | 15.0 (2.6)  14.1 (13.1, 16.8) | 0.044 | 13.6 (0.8)  13.7 (13.5, 13.9) | 13.9 (1.1)  13.8 (13.1, 14.4) | 0.542 |
| Values are expressed as mean (Standard Deviation) in the upper row and median (IQR) in the lower row.  P-values are calculated using the Mann-Whitney model.  CVD: cardiovascular disease NAD: Nicotinamide adenine dinucleotide NAM: nicotinamide Me-NAM: 1-methylnicotinamide 2-PY: 2-methyl-2-pyridone-5-carboxamide 4-PYR: 4-pyridone-3-carboxamide-1-β-D-ribonucleoside | | | | | | |
